# Evidence-based medicine training in general surgery in the United Kingdom: an exploratory snapshot survey study

**DOI:** 10.1007/s00423-025-03955-7

**Published:** 2025-12-18

**Authors:** Evripidis Tokidis, Saba P. Balasubramanian, Pirashanthie Vivekananda-Schmidt

**Affiliations:** 1 Division of Clinical Medicine, School of Medicine and Population Health, University of Sheffield, Sheffield, UK; 2 Doncaster and Bassetlaw NHS Foundation Trust, Doncaster, UK; 3 Sheffield Teaching Hospitals NHS Foundation Trust, Sheffield, UK

**Keywords:** Evidence-based medicine, Evidence-based surgery, General surgery, Attitudes, Barriers, Curriculum

## Abstract

**Purpose:**

This study aims to identify UK general surgical trainees’ perceptions, attitudes, and perceived barriers to EBM training and assessment so that interventions by general surgery educators to improve integration of EBM are informed by stakeholder data.

**Method:**

A mixed-method survey was developed by adapting the validated McColl and BARRIERS questionnaires, informed by a scoping review and focus group discussions. Ethical approval was obtained (University of Sheffield − 056808). The survey was distributed through social media, surgical society newsletters, and deanery mailing lists, adhering to the CHERRIES checklist.

**Results:**

The survey yielded 101 responses, 53 of which were complete (65% male, 35% female). A quarter of the 53 respondents did not hold higher academic degrees. Most participants (61%) worked in district general hospitals, with the highest response rates from Yorkshire and West Midlands. Attitudes towards EBM were predominantly positive from trainees (50.3%), with most of the respondents indicating their ability to understand and explain EBM terminology. However, they perceived their senior colleagues to be less enthusiastic about EBM (41.5%). Barriers to developing EBM competencies included lack of time, excessive evidence volume, limited access to resources, inadequate critical appraisal skills and limited opportunities for application during clinical practice. Existing postgraduate assessment strategies were deemed adequate for EBM by most of the trainees.

**Conclusion:**

The surveyed UK General surgical trainees exhibit positive attitudes towards EBM but face barriers in its application within their training. One way of addressing this issue is through research informed targeted curricular interventions.

**Supplementary Information:**

The online version contains supplementary material available at 10.1007/s00423-025-03955-7.

## Introduction

General surgical training is characterised by intensive clinical responsibilities and cultural norms that collectively shape the practice [1]. Within this environment, the routine integration of Evidence-Based Medicine (EBM) [2] defined as the “the conscientious, explicit, and judicious use of current best evidence in making decisions about the care of individual patients” remains elusive [3, 4]. Attitudes and barriers to EBM have been examined in the past using mixed method survey research which produced validated questionnaires (McColl questionnaire/BARRIERS scale) [5].

Although formally recognised as integral to modern surgical training [6], implementation of the principles of EBM is often overshadowed by the complexities of clinical service and professional socialisation, indicating that several factors prevent its adoption as demonstrated in recent work [3, 4]. In particular, a complex array of cultural, structural, and pedagogical barriers were revealed by the scoping review [4], while the focus group discussions with trainees further confirmed their systemic impact [3]. Interpreting these findings in isolation, however, risks overlooking subtle but crucial interconnections. Therefore, to achieve triangulation [7], the survey method [8] in addition to focus group discussions can help expand upon the previously identified themes with a larger group of stakeholders and thereby to better understand how to facilitate EBM Practice amongst trainee surgeons.

This survey aims to triangulate previous qualitative research findings by gaining a directional understanding of the factors limiting EBM uptake for surgical trainees and, in doing so, guide the creation of informed strategies that align with the realities of surgical education. The objectives include refining the conceptual model of these barriers developed through previous work [3, 4], identifying points for discussion with surgical education stakeholders to develop curricular reform, and ultimately advancing EBM integration within general surgical training.

## Methods

A mixed-methods survey [7] was undertaken to explore the perspectives of general surgery trainees in the United Kingdom on integrating EBM into their training. General Surgery trainees were selected as the primary participants due to their direct involvement in surgical education and their status as the primary beneficiaries of an EBM-focused curriculum. Surgeons not in an established general surgical training programme i.e. locally employed doctors [9], were excluded to maintain the survey’s relevance to its target audience.

A survey tool was developed (Survey_Tool) by adapting the McColl questionnaire [5] based on previous findings from a scoping review and focus group discussions from the research team [3, 4]. The draft questionnaire was piloted with ten general surgery trainees from different training grades. The pilot assessed clarity of wording, face validity, and feasibility of completion. Feedback over email or via in-person discussion led to minor revisions in the phrasing of items and streamlining of the layout, after which the survey was finalised by the authors. Pilot responses were used only to refine the instrument and were not included in the main analysis.

The survey was disseminated as an open invitation to general surgical trainees and was facilitated through professional societies, deaneries, and training networks, as the lead author did not have direct access to participants’ contact details. It is estimated that approximately 1,100 general surgical trainees work in the UK [10]. Key organisations, including the Association of Coloproctology of Great Britain and Ireland (ACPGBI) trainees club (The Dukes’ Club), and the Association of Surgeons of Great Britain and Ireland, distributed the survey via newsletters, mailing lists, and WhatsApp groups. A reminder was issued via the above communications to improve response rates. Several social media shares were issued to improve engagement. While efforts were made to engage all deaneries in the United Kingdom, not all participated in dissemination, which limited the ability to achieve full national coverage.

The raw data for this study was generated using Qualtrics^XM^ software (Copyright © 2025 Qualtrics. Qualtrics and all other Qualtrics product or service names are registered trademarks or trademarks of Qualtrics, Provo, UT, USA. https://www.qualtrics.com). Data were managed according to an established data management plan with the University of Sheffield (Ethics reference: 056808) and analysed using Microsoft Excel^™^ and Jamovi [11]. The CHERRIES (Checklist for Reporting Results of Internet E-Surveys) checklist [12] was adhered to throughout the design, dissemination, and reporting phases to enhance the survey’s rigour, transparency, and reproducibility. All participants provided electronic consent after reading a participant information leaflet to participate in the survey. To verify the participants, their General Medical Council (GMC) registration was requested.

The response rate (complete responses divided by all potential eligible cases, including an estimated fraction of unknown eligibles) and cooperation rate (the number of completed interviews divided by all contacted and confirmed eligible units) were calculated following the American Association for Public Opinion Research (AAPOR) standardised formulas [13].

All survey data were analysed using non-parametric methods appropriate for ordinal and non-normally distributed variables. Associations between trainee characteristics and survey outcomes were explored using the Kruskal–Wallis test. Attitude to EBM (5-point ordinal scale), confidence in EBM terminology (10 items on a 4-point Likert scale), and perceived barriers to EBM (15 items on a 4-point barrier-severity Likert scale) were compared across hospital type, training grade, and higher-degree possession. Free-text responses were analysed thematically and subsequently mapped to Kirkpatrick’s four-level framework [14] for training evaluation to provide an educational lens for categorisation.

National-level demographic data describing the general surgery trainee population by deanery, training grade, or hospital type are not publicly available. As such, it was not possible to assess the representativeness of respondents against the wider trainee cohort beyond the approximate total population estimate of trainees derived from GMC workforce reports. We therefore present detailed descriptive characteristics of the 53 respondents to allow readers to judge the distribution of the sample, while recognising that findings should be interpreted as indicative of this respondent group rather than generalisable to all UK trainees.

## Results

A total of 53 trainees returned a completed survey (4.8% response rate). Among the 101 trainees who clicked on the survey, those same 53 completed responses yield a cooperation rate of 52.4%. The survey respondents were predominantly male (65%), with a median age of 34.5 years (SD 3.89). A large number of participants were employed in district general hospitals (34/53, 64.2%), while 19/53 (35.8%) who worked in larger centres. Among the 53 complete responses, 11 trainees (20.8%) indicated they were out of programme for research. Of those in training, the distribution across training levels was as follows: ST3 − 14 (26.4%), ST4–5 (9.4%), ST5–9 (17.0%), ST6–3 (5.7%), ST7–4 (7.5%), and ST8–8 (15.1%). Approximately a quarter of respondents (24%) indicated that they did not have a doctoral degree such as an MD or PhD. Nevertheless, 35% of the respondents completed a Masters or Education degree.

Participants in the survey demonstrated an overall positive attitude towards EBM, as reflected in the results of the modified McColl questionnaire (Table 1). Of the 53 respondents, 24 (45.3%) indicated they were “extremely welcoming” towards EBM, while 16 (30.2%) described themselves as “somewhat welcoming.” A neutral attitude was reported by 11/53 respondents (20.8%). When asked about their perceptions of their fellow trainees’ attitudes towards EBM, participants reported similar sentiments to their own. However, when queried about their consultants’ attitudes, 22/53 respondents (41.5%) perceived these as either “neutral”, “somewhat unwelcoming” or “extremely unwelcoming”. Nevertheless, 32/53 trainees (60.4%) rated their consultants’ attitudes to EBM as “somewhat welcoming” or “extremely welcoming”. The attitudes were not associated with either the level of training/research background or hospital.

**Table 1:** Trainees’ attitudes of EBM terminology; compared against hospital type, level of training and higher degree qualifications. Data are presented number (n) and percentage (%) of respondents. The final three columns display p-values calculated using the Kruskal–Wallis test to assess differences across hospital type, level of training, and possession of a higher degree

Survey Question	Response distribution *n* (%)	Comparison across hospital type	Comparison across level of training	Comparison by higher degree
Do trainees welcome EBM learning and practice?	Extremely unwelcoming *n* = 1 (1.9%) Somewhat unwelcoming *n* = 2 (3.8%) Neutral *n* = 10 (18.9%) Somewhat welcoming *n* = 16 (30.2%) Extremely welcoming *n* = 24 (45.3%)	0.285	0.891	0.115
Do trainees feel that their fellow trainees welcome EBM learning and practice?	Extremely unwelcoming *n* = 1 (1.9%) Somewhat unwelcoming *n* = 5 (9.4%) Neutral *n* = 13 (24.5%) Somewhat welcoming *n* = 24 (45.3%) Extremely welcoming *n* = 10 (18.9%)	0.794	0.102	0.059
Do trainees feel consultants welcome EBM learning and practice?	Extremely unwelcoming *n* = 1 (1.9%) Somewhat unwelcoming *n* = 8 (15.1%) Neutral *n* = 12 (22.6%) Somewhat welcoming *n* = 21 (39.6%) Extremely welcoming *n* = 11 (20.8%)	0.297	0.415	0.334

The survey highlighted several barriers to EBM implementation (Table 2), with the most frequently reported being time constraints and the overwhelming volume of evidence, both cited as a moderate or major barrier by 39 of 53 trainees (73.6%). A lack of formal critical appraisal skills was similarly reported as a moderate or major barrier by 39 participants (73.6%), while complex statistics were perceived as a barrier by 37 (69.8%). Other commonly identified challenges included limited institutional support, difficulty accessing full-text resources, and the influence of surgical dogma. Distributions of barrier severity were broadly similar across hospital types, training grades, and academic backgrounds, with no statistically significant subgroup differences identified.

**Table 2:** Barriers to EBM; compared against hospital type, level of training and higher degree qualifications. Data are presented number (n) and percentage (%) of respondents. The final three columns display p-values calculated using the Kruskal–Wallis test to assess differences across hospital type, level of training, and possession of a higher degree

Barrier	Response distribution *n* (%)	Comparison across hospital type	Comparison across level of training	Comparison by higher degree
Lack of time	No barrier *n* = 2 (3.8%) Minor *n* = 10 (18.9%) Moderate *n* = 25 (47.2%) Major *n* = 16 (30.2%)	0.798	0.714	0.984
Complex Statistics	No barrier *n* = 3 (5.7%) Minor *n* = 11 (20.8%) Moderate *n* = 17 (32.1%) Major *n* = 22 (41.5%)	0.857	0.381	0.264
Lack of critical appraisal skills	No barrier *n* = 8 (15.1%) Minor *n* = 15 (28.3%) Moderate *n* = 14 (26.4%) Major *n* = 16 (30.2%)	0.951	0.468	0.729
Lack of good quality evidence	No barrier *n* = 5 (9.4%) Minor *n* = 18 (34.0%) Moderate *n* = 17 (32.1%) Major *n* = 13 (24.5%)	0.855	0.382	0.197
Access to EBM resources	No barrier *n* = 8 (15.1%) Minor *n* = 22 (41.5%) Moderate *n* = 14 (26.4%) Major *n* = 9 (17.0%)	0.191	0.862	0.844
Lack of investment by health authorities in EBM	No barrier *n* = 5 (9.4%) Minor *n* = 13 (24.5%) Moderate *n* = 19 (35.8%) Major *n* = 16 (30.2%)	0.303	0.142	0.858
No financial gain in using evidence-based medicine	No barrier *n* = 19 (35.8%) Minor *n* = 11 (20.8%) Moderate *n* = 16 (30.2%) Major *n* = 7 (13.2%)	0.860	0.405	0.370
Surgical Dogma	No barrier *n* = 7 (13.2%) Minor *n* = 8 (15.1%) Moderate *n* = 21 (39.6%) Major *n* = 17 (32.1%)	0.620	0.447	0.166
Evidence not related to context of clinical practice	No barrier *n* = 8 (15.1%) Minor *n* = 15 (28.3%) Moderate *n* = 22 (41.5%) Major *n* = 8 (15.1%)	0.982	0.510	0.567
Too much evidence	No barrier *n* = 11 (20.8%) Minor *n* = 17 (32.1%) Moderate *n* = 16 (30.2%) Major *n* = 9 (17.0%)	0.121	0.015	0.098
Availability and access to EBM resources	No barrier *n* = 7 (13.2%) Minor *n* = 20 (37.7%) Moderate *n* = 16 (30.2%) Major *n* = 10 (18.9%)	0.384	0.709	0.938
Patients’ expectations	No barrier *n* = 11 (20.8%) Minor *n* = 25 (47.2%) Moderate *n* = 10 (18.9%) Major *n* = 7 (13.2%)	0.333	0.554	0.352
Patients demanding ineffective treatment	No barrier *n* = 17 (32.1%) Minor *n* = 19 (35.8%) Moderate *n* = 9 (17.0%) Major *n* = 8 (15.1%)	0.674	0.730	0.496
The need for length discussions with patients	No barrier *n* = 16 (30.2%) Minor *n* = 18 (34.0%) Moderate *n* = 13 (24.5%) Major *n* = 6 (11.3%)	0.388	0.647	0.927
Attitudes of colleagues towards EBM	No barrier *n* = 8 (15.1%) Minor *n* = 18 (34.0%) Moderate *n* = 20 (37.7%) Major *n* = 7 (13.2%)	0.269	0.420	0.650

Most of the participants expressed confidence in their ability to understand and explain EBM terminology (Table 3). Despite this, there was variation in familiarity with specific EBM terms. “Odds ratio” exhibited the greatest variability in self-reported familiarity—respondents’ ratings spanned all four categories from “not helpful” through to “can explain to others”—whereas “systematic review” showed the least variability. Those respondents with an academic degree (MD/PhD) showed more confidence in explaining certain terms, particularly for systematic review (*p* = 0.026), odds ratio (*p* = 0.031), meta-analysis (*p* = 0.006), and publication bias (*p* = 0.017). Confidence in EBM terminology did not increase significantly with level of training for systematic review (*p* = 0.026), odds ratio (*p* = 0.031), meta-analysis (*p* = 0.006), and publication bias (*p* = 0.017).

**Table 3:** Trainees’ Understanding of EBM terminology; compared against hospital type, level of training and higher degree qualifications. Data are presented as number (n) and percentage (%) of respondents. The final three columns display p-values calculated using the Kruskal–Wallis test to assess differences across hospital type, level of training, and possession of a higher degree

EBM terminology	Response distribution *n* (%)	Comparison across hospital type	Comparison across level of training	Comparison by higher degree
Relative Risk	Do not understand and would like to *n* = 3 (5.7%) Some Understanding *n* = 27 (50.9%) Understand and could explain *n* = 23 (43.4%)	0.499	0.645	0.084
Absolute Risk	Do not understand and would like to *n* = 5 (9.4%) Some Understanding *n* = 23 (43.4%) Understand and could explain *n* = 25 (47.2%)	0.540	0.562	0.157
Systematic Review	Some Understanding *n* = 16 (30.2%) Understand and could explain *n* = 37 (69.8%)	0.096	0.464	0.026
Odds Ratio	Do not understand and would like to *n* = 7 (13.2%) Some Understanding *n* = 26 (49.1%) Understand and could explain 20 (37.7%)	0.820	0.267	0.031
Meta-Analysis	Do not understand and would like to 4 (7.5%) Some Understanding *n* = 17 (32.1%) Understand and could explain 32 (60.4%)	0.331	0.233	0.006
Clinical Effectiveness	Do not understand and would like to *n* = 7 (13.2%) Some Understanding *n* = 24 (45.3%) Understand and could explain *n* = 22 (41.5%)	0.487	0.738	0.437
Number Needed to Treat	Do not understand and would like to *n* = 3 (5.7%) Some Understanding *n* = 19 (35.8%) Understand and could explain *n* = 31 (58.5%)	0.330	0.253	0.554
Confidence Interval	Do not understand and would like to *n* = 3 (5.7%) Some Understanding *n* = 14 (26.4%) Understand and could explain *n* = 36 (67.9%)	0.821	0.538	0.356
Heterogeneity	Do not understand and would like to *n* = 2 (3.8%) Some Understanding *n* = 18 (34.0%) Understand and could explain *n* = 33 (62.3%)	0.230	0.636	0.135
Publication Bias	Do not understand and would like to *n* = 1 (1.9%) Some Understanding *n* = 15 (28.3%) Understand and could explain *n* = 37 (69.8%)	0.725	0.145	0.017

Survey responses showed strong but varied use of evidence-based resources. PubMed was used by 82% (with 68% applying them clinically), Cochrane Reviews by 71% (59% applying clinically), high-impact surgical journals by 88% (76% applying clinically), Specialty or Society Guidance by 80% (67% applying clinically), and recent surgical textbooks by 74% (52% applying clinically). Overall, 3–9% were unaware of Cochrane reviews and high-impact journals, while 14–20% were aware but had not used them.

Trainees’ perceptions of existing EBM training were mixed. Many respondents noted some exposure to EBM education through journal clubs, regional teaching sessions, and deanery-led workshops. However, these opportunities were not consistently available across all settings, and their perceived effectiveness varied among participants, indicating a need for more structured and universally accessible training methods. When asked how EBM training could be enhanced, participants suggested regional teaching sessions (18.9%), workshops (7.5%), and journal clubs (13.2%).

Free-text responses suggested a range of strategies for strengthening the integration of EBM into surgical training (Table 4). When mapped against Kirkpatrick’s framework, these spanned all four levels of training evaluation, from engaging delivery methods and structured teaching to embedding EBM in assessments and influencing policy at deanery and curriculum level. A large proportion of participants supported mandatory annual EBM training sessions, with many advocating for workshops on research methods and critical appraisal skills. Some respondents suggested incorporating EBM into work-based assessments (WBAs) within the ISCP framework. Adding an EBM-focused section to WBAs was proposed as a way to formalise EBM evaluation within regular trainee assessments.

**Table 4:** Suggestions of trainees categorised as per kirkpatric level of curricular development

Kirkpatrick Level	Themes	Example trainee suggestions
Level 1: Reaction (engagement & satisfaction)	Flexible, engaging delivery formats	Regional/national teaching programme with online delivery and flexible access
		Regional journal clubs online with annual research-themed teaching
		Live high-quality RCTs app/website with podcasts
Level 2: Learning (knowledge & skills acquisition)	Structured educational content	Mandatory annual teaching on research methods/critical appraisal
		Deanery training days focused on EBM
Level 3: Behaviour (application in practice)	Embedding into practice & assessment	Add EBM summary as a component of WBAs on ISCP
		Encourage involvement in collaborative research
Level 4: Results (organisational/system outcomes)	Policy & curricular change	Formalised delivery via deaneries
		Incorporate research requirements into CCT
		Lobby deaneries for stronger academic/EBM integration

## Discussion

This survey complements and corroborates the previous focus group study’s [3] conclusions on the current landscape of EBM within general surgical training in the United Kingdom. The survey captured perspectives from trainees at different hospital environments, levels of training and engagement with higher research degrees; hence supporting triangulation with earlier qualitative findings [3] and providing empirical direction for the design of a subsequent Delphi study. The key messages from the survey are that trainees generally hold positive attitudes towards EBM and recognise its value in improving surgical outcomes. However, barriers remain in embedding EBM into training; particularly in terms of applying EBM competencies in practice. This emphasises the need for targeted educational interventions to enhance its integration within the surgical curriculum.

The positive attitudes towards EBM observed are encouraging and suggest a growing acceptance of its principles across the surveyed trainees. Most respondents reported confidence in understanding and explaining EBM terminology (as provided by the McColl questionnaire [5]), reflecting a baseline level of familiarity with the terms examined. However, the variability reported in understanding specific terms might indicate inconsistencies in training due to differences in exposure and variable opportunities across training regions and hospital types. This supports the need for a more standardised and structured approach to EBM education, particularly at the postgraduate level [4, 15].

Most trainees reported regular use of online EBM resources. However, this reliance on self-directed resource use indicates that while trainees recognise the utility of these tools, formal training on their optimal use may not be uniformly provided across all training settings. It is important to note that the resources included in our survey were modified based on the McColl questionnaire [5], which does not have room for EBM training-specific resources or Evidence-based summaries [16].

The reported barriers to implementing EBM were multifaceted and reflected the systemic, cultural, and logistical challenges inherent in surgical training, in line with current literature [4]. Time constraints, overwhelming volume of evidence and lack of formal critical appraisal skills were the most notable barriers. Cultural factors, such as surgical dogma, even though prominent in previous qualitative work [3], were not perceived as significant barriers in this survey. This could be due to survey format being a limiting factor or it might reflect a trainee cultural shift not yet adopted by their senior colleagues.

Critical appraisal skills were identified as a key area for improvement, with many of the surveyed trainees acknowledging gaps in their ability to evaluate the quality and applicability of evidence. This aligns with previous research [15]. Addressing this deficit through general surgery specific educational interventions is therefore necessary [17].

Despite these challenges, the respondents – like other surveyed surgical trainees in the past [4] – expressed a desire for improved EBM education, emphasising the importance of regional and national initiatives. Suggestions included developing annual, deanery-led teaching programmes, establishing online asynchronous critical appraisal modules tailored to surgical cases. Additionally, some trainees suggested utilisation of existing journal clubs, and access to educational resources. Initiatives such as journal clubs are not uncommon [18] and previous studies report short term success of these interventions [19, 20], however long-term ‘knowledge decay’ was observed [21].

Furthermore, work-based learning [22] was identified as a potential assessment modality for EBM. Incorporating EBM-focused sections into existing portfolios [23] could provide trainees with regular opportunities to engage with evidence-based principles and reinforce their application in clinical practice. Additionally, education stakeholders can work together to clarify the extent of EBM in professional exams and existing CCT requirements.

## Limitations

The response rate is low. Though this is consistent with similar surveys of medical professionals [24]; it restricts the generalisability of the findings. Selection bias is also possible [25], as trainees with a pre-existing interest in EBM may have been more likely to participate; trainees with a greater interest or academic background in EBM may have been more likely to participate too, and the absence of national data on higher academic qualifications prevents contextualisation of this bias. As with all self-reported data, there is the possibility of response bias, whereby participants may have provided socially desirable rather than fully accurate responses though to minimise this issue, no personal information was collected. The geographical distribution of respondents was uneven, with some deaneries more strongly represented, which may affect applicability to regions with different training structures. It is however, reassuring that the survey conclusions corroborate with the previous focus group study [3] and the mix of methods across the two studies increase the confidence in the conclusions.

## Conclusion

This survey demonstrates that UK general surgery trainees who responded, report generally positive attitudes towards evidence-based medicine (EBM) yet face persistent barriers to its consistent application in training and practice. These include limited time, the volume of evidence, and variable appraisal skills. These limit opportunities to apply EBM competencies in clinical practice. While the low response rate constrains the generalisability of the findings, the survey adds value by offering a snapshot of these barriers and mapping confidence across key EBM terminology. When integrated with our prior research [3, 4], these results provide both breadth and depth, informing subsequent structured discussions with senior surgical education stakeholders. This sequential approach has aided the design of a Delphi consensus study aimed at generating actionable recommendations through a consensus curriculum for EBM competencies within general surgery in the UK.

## Supplementary Information

Below is the link to the electronic supplementary material.

Supplementary Material 1

Supplementary Material 2

## Data Availability

The data used to produce this review are available upon reasonable request to the corresponding author.
