# Supplementary material for: Evidence-based medicine training in general surgery in the United Kingdom: an exploratory snapshot survey study
Source: Langenbecks Arch Surg. 2025 Dec 18;411(1):51. doi: 10.1007/s00423-025-03955-7 (PMC12799627; doi:10.1007/s00423-025-03955-7)
Supplement: Supplementary file 1 — Supplementary Material 1 [file 423_2025_3955_MOESM1_ESM.docx]

This file presents the CHERRIES checklist (Checklist for Reporting Results of Internet E-Surveys) completed for this study. Adapted from: Eysenbach G. CHERRIES: Improving the quality of Web surveys: the Checklist for Reporting Results of Internet E-Surveys. J Med Internet Res 2004;6(3):e34.

| **Checklist item** | **Description (this study)** | **Location in manuscript** |
| --- | --- | --- |
| **Survey design** | Cross-sectional survey conducted using the Qualtrics online platform. | Methods, p.2-3 |
| **Ethics approval** | Approved by the University of Sheffield Research Ethics Committee. | Methods, p.2-3 |
| **Informed consent** | Participants were provided with detailed study information and gave electronic consent before participation. | Methods, p.2-3 |
| **Development and testing** | The questionnaire was internally piloted among surgical trainees and academic supervisors to ensure clarity and appropriate timing prior to dissemination. | Methods, p.2-3 |
| **Recruitment process** | Dissemination was conducted via professional surgical societies, deanery mailing lists, and social media (Twitter). Reminder messages were issued to maximise participation. | Methods, p.2-3 |
| **Advertising of the survey** | Survey invitations were distributed through national professional networks, including newsletters and online trainee forums. | Methods, p.2-3 |
| **Response rate** | The estimated overall response rate was 4.8%. Of 101 invitees who accessed the survey, 53 provided complete responses (cooperation rate 52.4%). | Results, p.8 |
| **Preventing multiple entries** | Qualtrics settings prevented duplicate responses by restricting entries via IP address and browser cookies. | Methods, p.2-3 |
| **Incentives** | No financial or material incentives were offered to participants. | Methods, p.2-3 |
| **Data protection** | All responses were anonymised. Data were stored securely on password-protected University of Sheffield servers accessible only to the research team. | Methods, p.2-3 |
| **Analysis** | Descriptive and non-parametric (Kruskal–Wallis) analyses were performed using Jamovi statistical software. | Methods, p.2-3 |
| **Handling of incomplete questionnaires** | Only fully completed questionnaires (n = 53) were included in the analysis; incomplete responses were excluded. | Methods, p.2-3 |
| **Open and closed questions** | The survey included a combination of Likert-scale (closed) and free-text (open) questions to capture quantitative and qualitative insights. | Methods, p.2-3 |
| **Statistical correction** | No weighting, imputation, or statistical correction was applied owing to the exploratory nature and sample size of the survey. Analyses were performed on complete cases only. | Methods, p.2-3 |
