# Supplementary material for: Evidence-based medicine training in general surgery in the United Kingdom: an exploratory snapshot survey study
Source: Langenbecks Arch Surg. 2025 Dec 18;411(1):51. doi: 10.1007/s00423-025-03955-7 (PMC12799627; doi:10.1007/s00423-025-03955-7)
Supplement: Supplementary file 2 — Supplementary Material 2 [file 423_2025_3955_MOESM2_ESM.docx]

EBM survey

Start of Block: Consent block

Evidence-based Medicine competency is considered essential for shared decision making in General Surgery; this is governed by the current training curriculum and is a certification criterion for surgeons who are training to specialise in General Surgery. In the UK, at present, there is no formal EBM educational programme for General Surgical trainees. This survey aims to: 1. To gain an understanding of perceptions and attitudes in relation to EBM amongst General Surgical trainees in the United Kingdom 2. To develop an insight into perceived barriers to achieving EBM competency and practice and potential solutions to address EBM training and integration into surgical training and practice.

Q1.1 Do you currently hold a national training number in the United Kingdom?

- Yes
- No

| 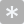 |
| --- |

Q1.2 This is a survey for General Surgery registrars currently working within a training programme; if you wish to provide feedback regarding your experience of EBM training in the UK please do so in the box below or by contacting the researcher on etokidis1@sheffield.ac.uk

________________________________________________________________

| Page Break |  |
| --- | --- |

Q1.3 Please read the participant information sheet

Q1.4


 By clicking the button below, you acknowledge the information on the participant information sheet and you agree to proceed with the survey. 

- I consent, begin the study
- I do not consent, I do not wish to participate

| Page Break |  |
| --- | --- |

Q1.5 Please enter your GMC number-we will not use this data as a personal identifier. This is only to avoid duplicate responses and responses from non-UK medically registered professionals. These will be destroyed at completion of survey data collection.

________________________________________________________________

End of Block: Consent block

Start of Block: Demographics

| 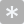 |
| --- |

Q2.1 My age is...

________________________________________________________________

| Page Break |  |
| --- | --- |

Q2.2 I describe my gender as...

- Female
- Male
- Non-Binary
- Genderfluid
- Intersex
- Prefer to describe
- Prefer not to say

| Page Break |  |
| --- | --- |

Q2.3 My training level is...

- ST3
- ST4
- ST5
- ST6
- ST7
- ST8
- Out of Programme-please specify if OOPT/OOPE/OOPC/OOPR/OOPP __________________________________________________
- Prefer not to say

| Page Break |  |
| --- | --- |

| 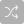 | 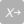 |
| --- | --- |

Q2.4 My training region is... (If you are currently Out of Programme, please choose the Deanery you hold a National Training Number in)

- East Midlands
- East of England
- KSS
- London
- Merseyside
- Northern
- Northern Ireland
- North Western
- Oxford
- Severn
- Scotland East
- Scotland North
- Scotland South East
- Scotland West
- South West and Peninsula
- Yorkshire and the Humber
- Wales
- Wessex
- West Midlands
- Prefer not to say

| Page Break |  |
| --- | --- |

| 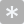 |
| --- |

Q2.5 Have you done (or are you currently undertaking) a post-graduate qualification?

- MD
- PhD
- Other-please specify __________________________________________________
- I do not hold any post-graduate qualification

| Page Break |  |
| --- | --- |

Q2.6 I currently work at...

- a District General Hospital
- a Tertiary Centre
- a Quaternary centre
- Prefer to specify __________________________________________________
- Prefer not to say

End of Block: Demographics

Start of Block: Attitudes to Evidence Based Medicine

Q3.1 Do you welcome EBM practice in your surgical training and place of work?

- Extremely unwelcoming
- Somewhat unwelcoming
- Neutral
- Somewhat welcoming
- Extremely welcoming

| Page Break |  |
| --- | --- |

Q3.2 How do you perceive your fellow trainees' attitude towards EBM?

- Extremely unwelcoming
- Somewhat unwelcoming
- Neutral
- Somewhat welcoming
- Extremely welcoming

| Page Break |  |
| --- | --- |

Q3.3 How do you perceive your consultant colleagues' attitude towards EBM?

- Extremely unwelcoming
- Somewhat unwelcoming
- Neutral
- Somewhat welcoming
- Extremely welcoming

| Page Break |  |
| --- | --- |

Q3.4 Do you agree that practice in EBM improves patient care?

- Completely disagree
- Disagree
- Neutral
- Agree
- Strongly agree

| Page Break |  |
| --- | --- |

Q3.5 Do you feel EBM is useful in day to day management of general surgical patients?

- Totally useless
- Somewhat useless
- Neutral
- Somewhat useful
- Extremely useful

| Page Break |  |
| --- | --- |

| 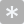 |
| --- |

Q3.6 Please provide an estimated (0-100) percentage (%) of your current practice that is evidence based

________________________________________________________________

| Page Break |  |
| --- | --- |

Q3.7 Please describe your understanding of the following EBM terms

|  | It would not be helpful for me to understand | Don't understand but would like to | Some understanding | Understand and could explain to others |
| --- | --- | --- | --- | --- |
| Relative risk |  |  |  |  |
| Absolute Risk |  |  |  |  |
| Systematic review |  |  |  |  |
| Odds ratio |  |  |  |  |
| Meta-analysis |  |  |  |  |
| Clinical effectiveness |  |  |  |  |
| Number needed to treat |  |  |  |  |
| Confidence interval |  |  |  |  |
| Heterogeneity |  |  |  |  |
| Publication bias |  |  |  |  |

| Page Break |  |
| --- | --- |

| Page Break |  |
| --- | --- |

Q3.8 Which of the following methods of moving towards Evidence Based Medicine **are you currently using?** (please select all that apply)

- Learning the skills of evidence based medicine i.e. identify and appraise literature or systematic reviews oneself
- Seeking and applying evidence based summaries which give the clinical 'bottom line'
- Using evidence based practice guidelines or protocols developed by colleagues for use by others

| Page Break |  |
| --- | --- |

Q3.9 Which of the following methods of moving towards Evidence Based Medicine **will you be using in the future?** (please select all that apply)

- Learning the skills of evidence based medicine i.e. identify and appraise literature or systematic reviews oneself
- Seeking and applying evidence based summaries which give the clinical 'bottom line'
- Using evidence based practice guidelines or protocols developed by colleagues for use by others

| Page Break |  |
| --- | --- |

Q3.10 Which of the following methods of moving towards Evidence Based Medicine **do you consider most appropriate in General Surgery?** (choose only one answer)

- Learning the skills of evidence based medicine i.e. identify and appraise literature or systematic reviews oneself
- Seeking and applying evidence based summaries which give the clinical 'bottom line'
- Using evidence based practice guidelines or protocols developed by colleagues for use by others

| Page Break |  |
| --- | --- |

Q3.11 Awareness of relevant EBM sources in General Surgery in the United Kingdom

|  | Unaware | Aware but not used/read | Use and/or read | Used to help in clinical decision making |
| --- | --- | --- | --- | --- |
| PubMed or other medical database |  |  |  |  |
| Cochrane Database of Systematic Reviews |  |  |  |  |
| High Impact surgery journals |  |  |  |  |
| Specialty or society guidance |  |  |  |  |
| Recent editions of surgical textbooks |  |  |  |  |

Q3.12 Any other EBM resources you are aware or using?

________________________________________________________________

End of Block: Attitudes to Evidence Based Medicine

Start of Block: Barriers to EBM implementation

Q4.1 To what extent do you perceive the following items as barriers to EBM practice

|  | No barrier | Minor | Moderate | Major |
| --- | --- | --- | --- | --- |
| Lack of time |  |  |  |  |
| Complex Statistics |  |  |  |  |
| Lack of critical appraisal skills |  |  |  |  |
| Lack of good quality evidence |  |  |  |  |
| Access to EBM resources |  |  |  |  |
| Lack of investment by health authorities in EBM |  |  |  |  |
| No financial gain in using evidence based medicine |  |  |  |  |
| Surgical Dogma |  |  |  |  |
| Evidence not related to context of clinical practice |  |  |  |  |
| Too much evidence |  |  |  |  |
| Availability and access to EBM resources |  |  |  |  |
| Patients' expectations |  |  |  |  |
| Patients demanding ineffective treatment |  |  |  |  |
| The need for lengthy discussions with patients |  |  |  |  |
| Attitudes of colleagues towards EBM |  |  |  |  |

| Page Break |  |
| --- | --- |

Q4.2 Any other barriers you prefer to describe?

________________________________________________________________

End of Block: Barriers to EBM implementation

Start of Block: Current EBM training in General Surgery in the United Kingdom

Q5.1 To what extent do you agree with the following statement?

|  | Completely disagree | Somewhat disagree | Neutral | Somewhat agree | Completely agree |
| --- | --- | --- | --- | --- | --- |
| I have had formal EBM training PRIOR to becoming a registrar on how to interpret evidence for my own practice |  |  |  |  |  |

Q5.2 To what extent do you agree with the following statement?

|  | Completely disagree | Somewhat disagree | Neutral | Somewhat agree | Completely agree |
| --- | --- | --- | --- | --- | --- |
| I have had formal EBM training AFTER becoming a registrar on how to interpret evidence for my own practice |  |  |  |  |  |

| Page Break |  |
| --- | --- |

Q5.3 Do you get any ***ongoing teaching or training*** in EBM in your current training programme?

- Yes
- No
- Unsure

| Page Break |  |
| --- | --- |

| 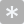 |
| --- |

Q5.4 If this is the case, how is EBM currently taught in your training programme?

- Embedded into regional teaching
- Deanery Workshops and/or courses
- Journal Clubs
- Ad-hoc lectures
- Prefer to describe __________________________________________________
- None of the above

| Page Break |  |
| --- | --- |

| 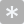 |
| --- |

Q5.5 Any suggestions of how training in EBM can be facilitated in current general surgical training?

________________________________________________________________

| Page Break |  |
| --- | --- |

Q5.6 Were you aware of the need to be assessed in your competence in EBM in order to complete your training?

- Yes
- No
- Unsure

| Page Break |  |
| --- | --- |

Q5.7 What do you feel is the most appropriate way of assessing EBM?  (can select more than one option)

- Annual Review of Competence Progression (ARCP)
- Fellowship of the Royal College of Surgeons (FRCS) exam
- Higher degree completion
- Publication in peer-reviewed journal
- ISCP portfolio assessment
- Audit
- Informal assessment
- Prefer not to say

| Page Break |  |
| --- | --- |

| 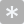 |
| --- |

Q5.8 Any suggestions of the assessment of EBM competence in current general surgical training?

________________________________________________________________

End of Block: Current EBM training in General Surgery in the United Kingdom
